# Inactivation in the potassium channel KcsA^☆^

**DOI:** 10.1016/j.yjsbx.2019.100009

**Published:** 2019-06-12

**Authors:** Yunyao Xu, Ann E. McDermott

**Affiliations:** Department of Chemistry, Columbia University, New York, NY 10027, United States

**Keywords:** Potassium channel, C-type inactivation, Solid state NMR, Allosteric coupling, KcsA

## Abstract

Inactivation, the slow cessation of transmission after activation, is a general feature of potassium channels. It is essential for their function, and malfunctions in inactivation leads to numerous pathologies. The detailed mechanism for the C-type inactivation, distinct from the N-type inactivation, remains an active area of investigation. Crystallography, computational simulations, and NMR have greatly enriched our understanding of the process. Here we review the major hypotheses regarding C-type inactivation, particularly focusing on the key role played by NMR studies of the prokaryotic potassium channel KcsA, which serves as a good model for voltage gated mammalian channels.

## Highlights

• C-type inactivation in potassium channels is a nearly universal regulatory mechanism.
• A major hypothesis states that C-type inactivation involves ion loss at the selectivity filter as an allosteric response to activation.
• NMR is used to probe protein conformational changes in response to pH and [K^+^], demonstrating that H^+^ and K^+^ binding are allosterically coupled in KcsA.
• The lipids are integrated parts of potassium channels in terms of structure, energetics and function.

## Graphical abstract

**Figure:**
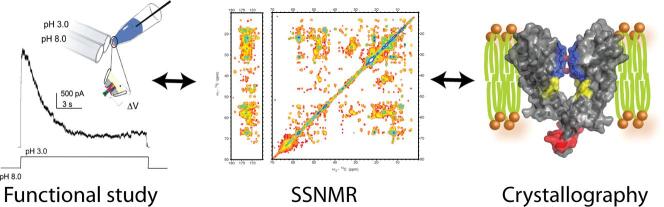

## 1 Introduction

Potassium channels form the second largest family of membrane proteins, and control numerous metabolic processes. Their primary function is to facilitate passive diffusion of potassium ions through low dielectric membrane, with great selectivity with respect to other ions (McCoy and Nimigean, 2012, Yellen, 2002). Ion channels play a wide variety of cell signaling roles in essentially all organisms, coupling this basic function to other environmental factors and cascading partners to form various complicated physiological functions (LeMasurier et al., 2001). As famously discovered by Huxley they play key roles in generating the membrane potential and its dynamical behaviour (Hodgkin and Huxley, 1952). In a wide array of organisms, including plants, bacteria, and even some viruses they play crucial roles in maintaining the cell potential which forms an energy reservoir and can be coupled to numerous other processes (Jentsch, 2000, Prindle et al., 2015). In excitable cells, such as neurons and muscles they control electrical conduction, taking advantage of the large concentration differences for sodium and potassium ions. In that context, potassium channels are typically activated following sodium channel-driven depolarization, and the potassium channels serve to recover the negative resting membrane potential and re-establish excitability (Hoshi and Armstrong, 2013).

Many diseases are connected to malfunction of potassium channels (Shieh et al., 2000). Roughly one hundred distinct potassium channels regulate a wide variety of critical CNS functions, including movement control and cardiac function, and therefore are drug targets for treatment in a wide range of disorders including vasodilation and blood pressure regulation, arrhythmias, neurodegenerative diseases and psychiatric treatments (Parekh et al., 2018). Deletion of one of the background potassium channels TREK-1 results in a depression-resistant phenotype (Heurteaux et al., 2006). Potassium channel mutations and malfunction (“channelopathies”) result in heart timing defects such as long QT syndrome (Ackerman, 2004). Another recent report shows that potassium channel expression level in cancer cells is notably higher than normal cells, causing a 2–3 fold lower intracellular potassium concentration. This destabilizes the K^+^ stabilized G-quadruplex structure, which is an important transcription factor for cancer inhibition (Tateishi-Karimata et al., 2018).

**Fig. 1:**
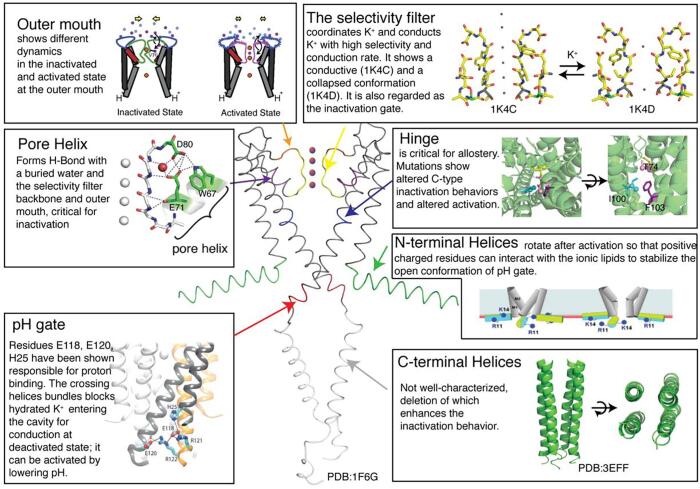
Overview of the structure of homo tetrameric KcsA and the reported functions. The protein has been dissected into the outer month (Raghuraman et al., 2014), the selectivity filter (Zhou et al., 2001), the pore helix (Bhate and McDermott, 2012), the hinge, the N-terminal helices (Iwamoto and Oiki, n.d.), the pH gate (Thompson et al., 2008) and the C-terminal helices (Uysal et al., 2009).

**Fig. 2:**
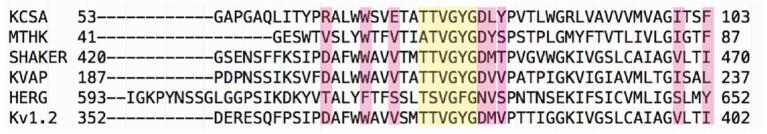
Sequence alignment of KcsA and other potassium channels. The stretch of residues highlighted in yellow is the most conserved region, and is termed the selectivity filter because of its involvement in binding and conduction of K^+^ ions. Residues highlighted in pink have been shown to be critical for the inactivation in KcsA.

Because of the significance of potassium channels in physiological functions, they have been the subjects of numerous structural and functional studies (Bhate and McDermott, 2012, Chakrapani et al., 2007b, Devaraneni et al., 2013, Gao et al., 2005, Hoshi et al., 1990, Hoshi and Armstrong, 2013, MacKinnon, 2003, Pan et al., 2011, Thomson and Rothberg, 2010). As suggested by strong sequence conservation (Yifrach and MacKinnon, 2002, Zhou et al., 2001), the pore domain structure, discovered in the landmark work on KcsA (Fig. 1) is well conserved in the K^+^ channel family. KcsA is a prokaryotic channel (LeMasurier et al., 2001) that has served as a much needed “user friendly” model system for potassium channel research. In the prototypical pore domain, two transmembrane helices (LeMasurier et al., 2001) and an extensive extracellular linker form a homo-tetramer. The extracellular selectivity filter, the locus of transmission of ions, is formed at this tetramer interface, with several well-crafted ion binding sites in a single file arrangement, each binding site containing ligands from all four monomers. The monomers also cross at the intracellular side to form an iris-like controlled steric block that is believed to serve as the activation gate for most channels. A wide diversity of mechanisms control this gate, frequently ligands or voltage or even both in combination. KcsA has a pH-controlled gate that has been characterized in detail (Liu et al., 2001, Thompson et al., 2008). More broadly, potassium channels exhibit also some variability in their structures. For example, most potassium channels are tetrameric, but some are dimeric; for each monomer, some have six transmembrane helixes, but some have two (Yellen, 2002). There are a variety of regulatory and activation control mechanisms (Kaczmarek et al., 2017, Thomson and Rothberg, 2010, Yellen, 2002). In spite of the diversity, they all share a structural motif and are conserved in key portions of their sequences (Fig. 2) (Yifrach and MacKinnon, 2002, Zhou et al., 2001).

Even though each potassium channel has its own ‘personality’, the research to understand potassium channel can be generally divided into three interdependent aspects that pertain to essentially all channels: permeation, gating, and inactivation (Cuello et al., 2010a, MacKinnon, 2003). We suggest recent reviews on permeation and gating (McCoy and Nimigean, 2012, Noskov and Roux, 2007, Swartz, 2004), and focus here on inactivation (Baukrowitz and Yellen, 1996, Cheng et al., 2011, Cuello et al., 2010a, Hoshi et al., 1990, Weingarth et al., 2014, Xu et al., 2017).

Remarkably, potassium channels can simultaneously achieve high selectivity and a high permeation rate. Potassium channels are selective for K^+^ over other ions such as Na^+^ by more than three orders of magnitude (Doyle, 1998). This selectivity is crucial considering the complicated intracellular environments. On the other hand, potassium channels can conduct K^+^ at a rate of 10^7^–10^8^ ions/second, close to the diffusion limit (Morais-Cabral et al., 2001, Yellen, 2002). These two properties seem contradictory from a naive point of view. The rapid conduction suggests high k_on_ and high k_off_ rate. The good selectivity would normally suggest high affinity, and therefore slow k_off_ rates. The high-resolution crystal structure of KcsA and in particular the selectivity filter suggest a solution to this question (Doyle, 1998, Zhou et al., 2001) (Fig. 1). The backbone carbonyls of the residues in the selectivity filter arrange themselves to coordinate K^+^ with their oxygen atoms in a 4-site single file structure. Hopping between sites is at a minimal energy cost, and if the barriers between the sites are similarly low, the permeation can therefore be quite fast (Zhou et al., 2001). Meanwhile, interactions between K^+^ and the selectivity filter compensate the dehydration energy of potassium ions; therefore, potassium ions can enter the selectivity filter with minimal energy barriers. This is by contrast with sodium ion, with a smaller radius higher dehydration energy and lower binding energy in the selectivity filter. Other factors such as induced electronic repulsion between pore loops when sodium ions are coordinated could also contribute to the channel selectivity (Åqvist et al., 2000, Berneche and Roux, 2002, Noskov and Roux, 2007). For summaries of mechanisms of channel selectivity and high conductivity, recent reviews are recommended (McCoy and Nimigean, 2012, Sansom et al., 2002, Varma et al., 2011).

Although the selectivity filter structure is highly conserved for almost all potassium channels, channels differ considerably in their activation processes, in terms of the stimuli ligands (proton, calcium ion and nucleotide) or voltage and the mechanisms (Bezanilla et al., 1991, Gao et al., 2005, Ye et al., 2006). In the C-terminus, transmembrane helices are bundled together to prevent hydrated ions (∼8 Å) from entering the channels, forming the activation gate (Bezanilla et al., 1991, Liu et al., 2001, Shimizu et al., 2008). These residues are sensitive to voltage or ligand binding; when activated they induce movement of helices to open the channel (Jensen et al., 2012, Sand et al., 2013). KcsA is a proton-activated channel and residues such as H25 E118, E120, are protonated during activation to disrupt the original hydrogen-bonding network, which holds the helices bundle, thus opens the pH gate (Imai et al., 2010, Thompson et al., 2008) (Fig. 1). The membrane has been reported to stabilize the open conformation by interacting with positively charged side chains of the N-terminal helices (Iwamoto and Oiki, 2013a, Iwamoto and Oiki, 2013b). Activation occurs on a faster dynamic timescale in the majority of potassium channels compared to the inactivation process, which we will discuss in the next section; in KcsA, the activation rate is in the range of 10^1^–10^2^ s^−1^. However, a recent study by Heer et al. argues that the selectivity filter might also play a critical role in the activation: through molecular dynamic simulation and electrophysiological study on a mutant, they implied that opening the pH gate can relax the selectivity filter and cause a loose affinity towards K^+^, which leads to ion conducting; they reasoned that the high K^+^ affinity at the selectivity filter could lead to a high activation energy for ion hopping (Heer et al., 2017).

## 2 Inactivation

**Fig. 3:**
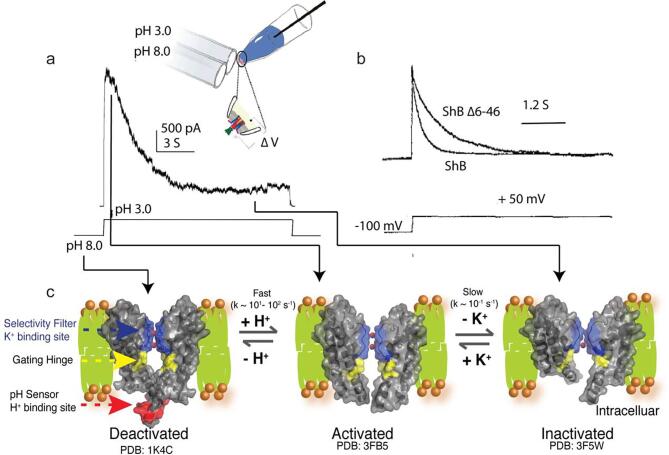
Inactivation of potassium channels. (a) The macroscopic current of KcsA in the patch-clamp measurement. KcsA is a pH-gated potassium channel. After activation by a pH drop from pH 8.0 to 3.0, the channel inactivates spontaneously and the current decreases gradually (b) The macroscopic current of Shaker measured by the patch-clamp technique. Shaker is a voltage-gated potassium channel, activated by switching the voltage from −100 to 50 mV. Shaker has two inactivation processes, the fast-decay current for the N-type inactivation and the slow-decay current for the C-type inactivation after the residues 6–46 were deleted. Adapted from reference (Sudha Hoshi et al., 1991). (c) Activation-coupled inactivation mechanism shown in KcsA. At pH 8.0, the channel is in the deactivated state; the activation gate is closed and the selectivity filter is in the conductive conformation. After the pH is dropped to 3.0, the channel converts to the activated state and both the activation gate and the selectivity filter are in the open conformation; potassium ions flow through the channel. The opening of the pH gate is also slowly coupled to the selectivity filter and biases the collapsed conformation. Therefore, the current decreases gradually until it reaches a plateau with minimal conductance.

By contrast with activation, inactivation is a process to close the channel in the sense of cessation of flow of ions. Inactivation is distinct from the reverse process of activation: the stimuli that activate the channel are still present, but the channels no longer conduct ions efficiently (Cuello et al., 2010b, Kurata and Fedida, 2006). In almost all potassium channels and many other ion channels, inactivation starts spontaneously as a consequence of activation, manifested as a decrease in current until it reaches a plateau with minimal conductance (Hoshi et al., 1991) (Fig. 3a,b). Two major types of inactivation were identified in the Shaker channel, a voltage-gated bacterial potassium channel (Hoshi et al., 1990). One is called N-type inactivation. It has a fast timescale and is caused by the positive N-terminus entering the channel cavity and blocking potassium ion conducting pathway (Molina et al., 2008). After deleting N-terminal residues, a slow inactivation process, normally on a seconds timescale, was discovered and named C-type inactivation (Choi et al., 1991, Hoshi et al., 1990). KcsA does not exhibit N-type inactivation, but it inactivates in a similar way to the C-type inactivation in Shaker and other voltage-gated potassium channels (Cuello et al., 2010b). C-type inactivation is crucial for regulating the physiological potassium ion concentration kinetically, and to control the firing frequency and duration of excitable cells (Hoshi and Armstrong, 2013, Rasmusson et al., 1998). Malfunction of the process is related many arrhythmias, including long QT syndrome (Burgess et al., 2012, Jentsch, 2000). Below where we refer to “inactivation”, it is understood to be C-type inactivation unless otherwise specified.

Numerous studies using molecular biology and characterization of site specific mutants, electrophysiology, crystallography, EPR, NMR, molecular simulation, ITC and other methods have focused on studying the mechanism of C-type inactivation (Ader et al., 2009, Bhate et al., 2010, Chakrapani et al., 2007a, Cordero-Morales et al., 2006, Devaraneni et al., 2013, Imai et al., 2010, Kim et al., 2016, Maffeo et al., 2012, Pan et al., 2011, Piasta et al., 2011, Tilegenova et al., 2017, Varga et al., 2007, Wang and MacKinnon, 2017). A consensus appears that the inactivation process is mainly related to the selectivity filter. This conclusion was based on several facts: firstly, many mutations around the selectivity filter modulate C-type inactivation process (Cordero-Morales et al., 2007, Kiss et al., 1999); secondly, the concentration of permeating ion and type of permeating ion, such as Rb^+^ strongly affect the inactivation process (Swenson and Armstrong, 1981); thirdly, many channels, after inactivation, show different ion selectivity (Kurata and Fedida, 2006). Thus, the selectivity filter is also referred as the inactivation gate. However, much debate still exists surrounding the molecular details of the conformational changes at the selectivity filter to lead to inactivation, and various hypotheses have been proposed based on different experimental results.

Here we review three main hypotheses surrounding the mechanism of C-type inactivation. The first two hypotheses are mainly discussed in the context of KcsA, even though some effects are shared with voltage-gated channels. The last hypothesis is mainly discussed in the context of voltage-gated channels. KcsA has been extensively used as a model for other mammalian potassium channels in term of channel activation and selectivity due to the stability of protein and ease of sample prepare and availability of various crystal structures. It is an interesting question to ask whether it is also a good model for inactivation and we will come back on this question later. We describe experimental efforts towards elucidation of the inactivation mechanism by NMR from our laboratory and others’.

### 2.1 Activation-coupled inactivation hypothesis

In the activation-coupled inactivation hypothesis, the inactivation process involves both the selectivity filter and the activation gate (Cuello et al., 2010b). The opening of the activation gate is dual-functional: it not only allows the ions to enter the channel for conducting, but also leads directly (although slowly) to an inactivation deformation at the selectivity filter via an allosteric coupling. In KcsA, activation and channel opening via protonation occurs on a millisecond timescale, while the inactivation process occurs on the timescale of seconds; therefore, ions can be permeated through the membrane in a controlled fashion for a window of time. This hypothesis can explain the macroscopic electrophysiological study in great detail. When the channel is in the deactivated or resting state, i.e. when the activation gate is closed and the selectivity filter is in the conducting form, there is no current. After the channel is activated by stimuli, such as a pH change for KcsA, the activation gate is opened on the millisecond-scale and the selectivity filter is still in the conductive conformation, thus there is current going through the membrane. However, the opening of the activation gate allosterically induces the collapsed state of the selectivity filter. Therefore, the selectivity filter enters the collapsed conformation on a seconds timescale. The current drops accordingly until it reaches a low-conducting plateau (Fig. 3c).

A primary question regarding this hypothesis has been the structure of the non-conducting form of the selectivity filter. MacKinnon and coworkers published two crystal structures of KcsA that show two conformations of the selectivity filter: the conductive form and the collapsed form. They were crystalized in a relatively high (200 mM) and low (3 mM) potassium concentration buffer, respectively, at neutral pH (Zhou et al., 2001) (Fig. 1). Even though the collapsed conformation was not crystalized under inactivating conditions, it has been hypothesized to represent the non-conducting conformation in the inactivation process.

Many experimental studies support this assumption, showing that perturbations causing less inactivation stabilize the conductive structure (Cuello et al., 2010a, Hoshi et al., 1990). Electrophysiology studies show that the kinetics of inactivation are dependent on permeant ions; a high K^+^ concentration slows the inactivation rate (Ostmeyer et al., 2013). Much evidence shows that a high K^+^ concentration stabilizes the conductive conformation (Xu et al., 2017, Zhou et al., 2001). Other permeant ions, like Rb^+^ also decelerate the inactivation; they are hypothesized to prevent the selectivity filter from collapsing by a longer residence time in the selectivity filter due to their slightly larger radius, which is known as ‘a foot at the door’ mechanism (Kurata and Fedida, 2006). Cordero-Morales et al showed that disruption of the hydrogen-bonding network between residues in the selectivity filter and its adjacent pore helix by mutating the E71 residue to alanine abolished the inactivation (Cordero-Morales et al., 2007) (Fig. 1). Crystallographic studies showed that the collapsed structure of the E71A mutant was not observed. NMR potassium affinity measurement revealed that E71A remains in a conductive conformation at low pH condition with low potassium concentration, further confirming that the collapsed conformation observed in the WT channel is significantly destabilized in E71A (Bhate and McDermott, 2012).

**Fig. 4:**
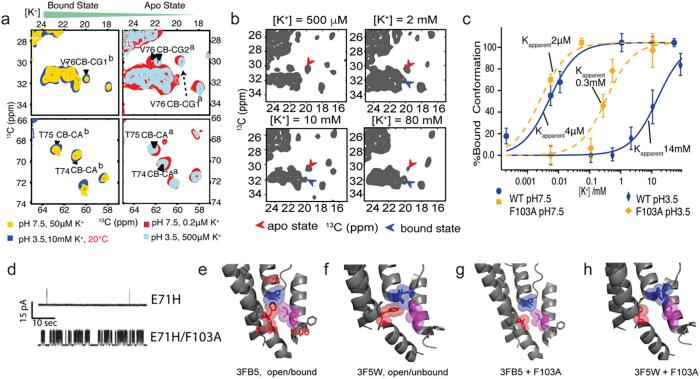
NMR chemical shifts indicate conformational changes and a potassium affinity change at the selectivity filter. (a) Similar chemical shift changes imply similar structural transitions for the selectivity filter of KcsA, regardless of pH, as indicated by the ^13^C–^13^C marker peaks of residues at the selectivity filter (e.g., T74, T75, V76). Presumably these structural changes correspond to conformational change between the conductive and collapsed selective filter seen in crystal structures. (b) Population of the K^+^-apo (collapsed) and K^+^-bound (conductive) states in the structural transitions modulated by ambient [K^+^]. (c) Titration experiments show that the potassium affinity changes by more than three orders of magnitude from 4 ± 1 μM for pH 7.5 and 14 ± 1 mM at pH 3.5. This indicates that the pH gate and the selectivity filter is strongly coupled at WT-KcsA. While similar measurements on the mutant F103A, in which C-type inactivation is greatly suppressed, a much smaller affinity change is measured, indicating a reduced coupling network. (d) Single channel recording experiments show that F103A can rescue the enhanced C-type inactivation caused by E71H, thus F103A reduces inactivation. (e–h) Structural models demonstrating the reduced coupling in F103A. The smaller side chain of alanine reduces the steric contact with neighbor residues such as T74 and I100. Adapted from the literature (Cuello et al., 2010b, Xu et al., 2017).

In other NMR studies WT-KcsA samples were prepared in various conditions, including pH 3.5 and 7.5 with low and saturated [K^+^] (Xu et al., 2017). Experimental results showed that two sets of chemical shifts existed for the selectivity filter, in accordance with the two conformations seen in the crystal structures. (Fig. 4a) Notably, the spectra of the selectivity filter displayed in the sample prepared in an inactivation conditions (pH 3.5 and low [K^+^]) are highly well overlaid with the spectra of the sample prepared at neutral pH and low [K^+^], implying that the collapsed and K^+^-depleted structure may actually be a good model for the inactivated state.

**Fig. 5:**
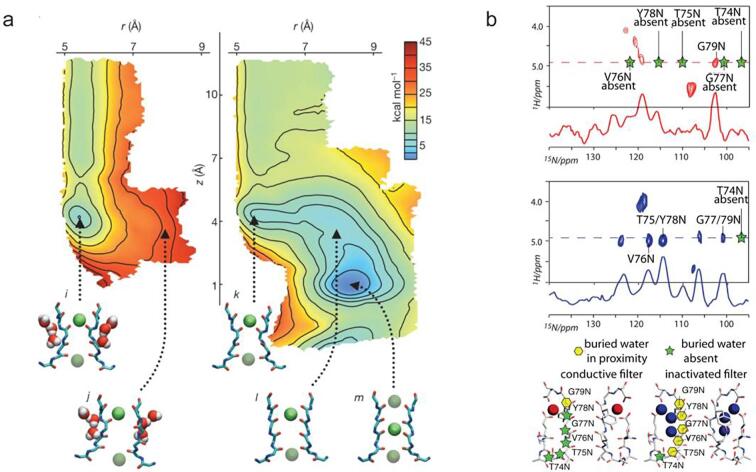
Buried water molecule(s) are critical to stabilize the collapsed conformation during inactivation, as shown by computational and SSNMR method. (a) Potential force calculation shows that when the water molecules are buried behind the selectivity filter, there is a high energy barrier to move K^+^ ion into the S2 site and convert the selectivity filter into a conductive conformation. However, when these waters are removed, the energy barrier for the transition is reduced. (b) SSNMR study confirms that there are buried water molecules behind the selectivity filter in the collapsed state, which are not present in the conductive conformation. Taken from reference (Ostmeyer et al., 2013, Weingarth et al., 2014).

The buried water molecules behind the selectivity filter have been hypothesized to be critical to stabilize the collapsed conformation in the inactivation state and potential of mean force calculations show that removal of the buried water affect the recovery process of inactivation, which takes up to several seconds (Ostmeyer et al., 2013) (Fig. 5a). Weingarth et al successfully detected the different water molecules distribution behind the selectivity for the collapsed and conductive conformation by SSNMR (Weingarth et al., 2014), echoing the finding in the computational calculations (Fig. 5b). Further experimental test aiming to accelerate the buried water molecule release in the collapsed conformation by increasing osmotic stress did increase the recovery rate from inactivation state (Ostmeyer et al., 2013), indicating that the collapsed conformation is the structure in the inactivation state.

Another question regarding the activation coupled inactivation hypothesis is how to demonstrate directly the coupling of the two gates. The selectivity filter is located on the extracellular side of the membrane, while the activation gate is located on the intracellular side. The distance between these two gates is about 30 Å. Such long distance communication is quite frequently observed in biology and referred as ‘allosteric coupling’ (Nussinov et al., 2014). Several strategies have been used in order to demonstrate transmembrane allosteric coupling in this system. In the first strategy, one of the gates can be specifically perturbed, and the response in the other gate is monitored. Several studies have measured the potassium affinity at the selectivity filter as a function of pH to test the allostery. Shimada et al measured potassium affinity for KcsA embedded in detergent micelle at pH 3.2 and pH 6.6, showing a 10-fold decrease in affinity at pH 3.2 (Imai et al., 2010). SSNMR measurements of KcsA prepared in lipid bilayer at pH 3.5 and pH 7.5, by contrast demonstrated that proton binding at the pH sensors caused more than three orders of magnitude change in potassium affinity (Fig. 4b,c) (Xu et al., 2017). The reduced potassium affinity after the pH gate activation can well explain the decreasing current during inactivation since potassium binding at the selectivity filter is required for ion conducting. The discrepancy in the coupling strength between solution NMR and SSNMR could be due to the experimental conditions: in solution NMR detergent micelles were used and the measurement was done on a C-terminus truncated KcsA at relatively high temperature (i.e. 45 °C), but in solid state NMR liposomes were used and the experimental temperature was done on a full-length KcsA at around 0 °C (Imai et al., 2010, Xu et al., 2017). The usage of detergent micelles could potentially alter the structure and dynamics of membrane protein due to the considerable change in exposure of protein to aqueous environment, lateral pressure and protein-lipid interactions, which has been documented in several cases (Chipot et al., 2018). It was also suggested that in the detergent micelles the pH gate of C-terminus truncated KcsA tends to be in an open conformation at pH 7, rendering a smaller coupling energy between the pH gate and the selectivity filter (Liu et al., 2015).

**Fig. 6:**
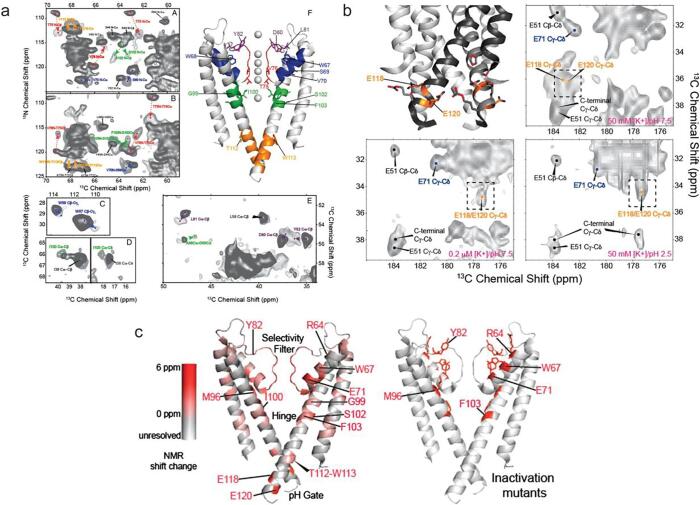
(a) NMR chemical shifts monitor a global structural change during the K^+^-apo (collapsed) and K^+^-bound (conductive) transition in response to ambient [K^+^] change. (b) NMR chemical shifts report \ on change of protonation state in the pH sensor in response to pH change. The pH sensor also gets protonated at pH 7.5, 0.2 μM [K^+^], indicating a shifted pKa caused by the coupling to the selectivity filter. Low ambient [K^+^] collapses the selectivity filter, which favors an open pH gate. (c) Residues of known functional importance display large chemical shift perturbations between conformational changes. This correlation demonstrates the power of NMR to study the channel conformal change and allostery. Taken from reference (Wylie et al., 2014).

What is the identity of the titratable residue coupled to K^+^ binding in the selectivity filter? The proximal acid E71, which had a confirmed role in inactivation, is a candidate. Also two acids in the pH sensor (E118 and E120) are candidates. The nature of the coupled acid being the pH sensor residues was confirmed with NMR studies, monitoring the protonation of the pH sensor as the K^+^ was titrated at constant pH and ionic strength (Wylie et al., 2014). Although changes in hydrogen bonding caused relatively minor changes in the NMR spectrum of E71 as the [K^+^] was changed, the changes in the spectra of the pH sensor residues were consistent with full protonation (Bhate and McDermott, 2012). Besides these residues, additional residues also show chemical shift changes, indicating a global structure change as ambient [K^+^] changes. (Fig. 6a) These residues show a high overlay with residues displaying functional importance. (Fig. 6c) Furthermore, a mutant in the pH sensor, H25R&E118A, has been characterized by SSNMR as well. These mutations render the channel constitutively open at the pH gate (Posson et al., 2013). As expected, the selectivity filer exhibits notably weak binding for K^+^ ions.

Analogously, SSNMR studies have indicated that the pKa of the proton binding residue at the activation gate is dependent on the conformation of the selectivity filter. The relative protonation of E118/120 is known to be influenced by both pH and ambient [K^+^] (Ader et al., 2009). More specifically the collapsed selectivity filter is induced by low ambient [K^+^], and the collapsed filter stabilizes the open activated gate, shifting the pKa of E118/E120 to above 7.5 (Fig. 6b) (Wylie et al., 2014). The pH gate opening was also monitored at various conditions by EPR studies, reporting a 1 pH unit pKa shift between the transition from deactivated to inactivated state and the reverse transition, suggesting that different intermediate states are involved in these two processes and the proton binding and that pH gate opening is coupled with the potassium binding (Tilegenova et al., 2017). When perturbations that inhibit C-type inactivation were introduced, such as mutating E71 to alanine, substituting Cs^+^ or Rb^+^ for K^+^, the observed pKa shift was diminished, supporting the conclusion that such a coupling is closely related to inactivation.

**Fig. 7:**
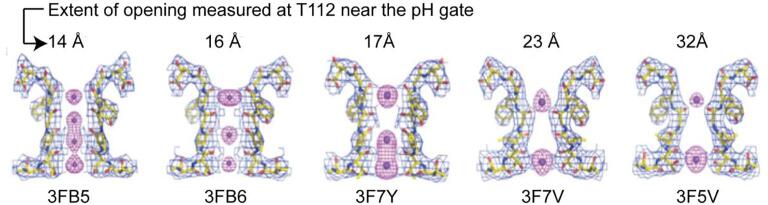
Crystal structures show that the extent of opening at the pH gate is coupled to the conformation and ion binding at the selectivity filter. Adapted from reference (Cuello et al., 2010a).

The determination of a series of KcsA crystal structures with open activation gates provided further direct support for the coupling mechanism (Cuello et al., 2010b, Cuello et al., 2010a). In this study, the pH gate was mutated to bias it to remain open, in order to mimic the activated and inactivated state. Various extents of opening at the activation gate were observed (in heterogeneous crystals), forming a kind of reaction coordinate. A key result of the study is that the degree of opening was correlated to conformation and ion binding at the selectivity filter: large opening at the activation gate was associated with more collapsed conformations and less ion binding at the selectivity filter (Fig. 7).

In a second strategy, residues between these two gates were perturbed to see if the coupling is attenuated or abolished. Several residues were revealed to be vital for the coupling between these two gates, many of which are located between the selectivity filter and the activation gate. For example, F103′s bulky aromatic side chain displays two torsion angles, correlating to the conductive and collapsed conformation. Mutation to a smaller sidechain residue such as alanine greatly reduces the C-type inactivation; in mutations to other residues with bulky sidechain such as Tyr, C-type inactivation is not affected (Cuello et al., 2010a). It is hypothesized that the bulky sidechain in F103 interacts with I100 and T74 to mediate the coupling from activation gate to the selectivity filter. We explored this hypothesis and find that protonation at the pH gate for an F103A mutant has a much weaker effect on potassium affinity at the selectivity filter than in the WT-KcsA (2uM to 300uM for F103A comparing to 4uM to 14 mM for WT-KcsA), suggesting that the coupling strength was perturbed by the mutant (Fig. 4c–h). Functional tests on I100 by electrophysiology also show that mutating I100 to alanine weakens the inactivation (Pan et al., 2011). This coupling network was further teased apart using NMR based thermodynamic measurements and single channel recordings to show that removal of T74 Cg sidechain in the T74S mutant greatly abolishes the pH effect on the potassium affinity and by extrapolation the inactivation of this mutant (Xu et al., 2019). All of these mutations are in a location near the cavity, distant from the pH gate and the selectivity filter; their effects on the coupling strength and inactivation behaviors offer a strong support for the hypothesis that inactivation involves coupling between the pH gate and the selectivity filter and clarify the participants in this coupling network.

Despite the apparently clear picture from the results above, this hypothesis was challenged by Valiyaveetil and coworkers based on experimental results on semi-synthesized KcsA channels (Devaraneni et al., 2013). These authors introduced an unnatural amino acid D-alanine at the selectivity filter to replace a glycine at Gly77. This substitution restricts the rotation of Gly77-Val76 peptide bond and prevents the selectivity filter entering the collapsed state. Its crystal structure was collected at 1 mM [K^+^] and the selectivity filter stays in the conductive form, in contract to a collapsed structure for WT at a similar condition (i.e. 3 mM [K^+^]). However, this structural modification did not cause changes the inactivation behavior of KcsA, challenging the hypothesis that the collapsed conformation represents the selectivity filter in the C-type inactivation. These studies were also performed on the voltage-gated KvAP channel and similar results were achieved. A recent computational study by Li et al examined this study in more detail, reporting that KcsAD-Ala77 channel could adopt an asymmetrical constricted-like nonconductive conformation in a molecular dynamics simulation (Li et al., 2017). Perhaps the chemical substitution or difference in crystallization conditions might account for the fact that the collapsed structure was not seen at 1 mM [K^+^] for KcsAD-Ala77 as they shifted K^+^ K_d_ to a tighter binding relative to WT. NMR measurements have indicated that the potassium affinity is very tight, around 4–10 μM in lipid bilayers at neutral pH, much lower than the 1 mM concentration (Bhate et al., 2010). These crystal structures were acquired with a closed activation gate; whether the selectivity filter adopts a conductive conformation or not after activation is unknown.

New perspectives for the inactivation process emerge from computational studies, which have been recently reviewed from a methods development perspective (Harpole and Delemotte, 2018). Recent MD studies explored the mechanism of the inactivation process (Li et al., 2018), with microsecond MD simulations and replica-exchange umbrella sampling. This study concluded that the semi-open state (3FB5 in Fig. 7) is stable but truncated KcsA in the fully open Activated state relaxes to form a “pinched” selectivity filter within 1–2 μs. Since the microsecond timescale is too fast to be the rate-determining step of inactivation, it was further suggested that the semi-open to open transition is the rate-determining step of inactivation. This picture raises the broader question of the process of activation. One study used Essential Dynamics (Amadei et al., 1996) and umbrella sampling to study the process of activation (Linder et al., 2013), identifying a low barrier in the initial steps of activation including a rotamer flip in conserved F114 of TM2; subsequent global rearrangements were predicted to be slower. Another MD study (Heer et al., 2017) indicated that changes in the channel’s shape during activation leads to important dynamical changes in the selectivity filter, triggering increased ion permeation. In other words, allosteric dynamical events in KcsA’s selectivity filter function as a dynamical switch, offering a possible explanation for the fact that some ligand-gated K^+^ channels apparently lack an activation gate. The relation of these activation events to inactivation is not fully clear. Computational studies and integrated computations and experiments hold great promise for providing a much more solid explanation for inactivation.

### 2.2 Ion binding hypothesis

**Fig. 8:**
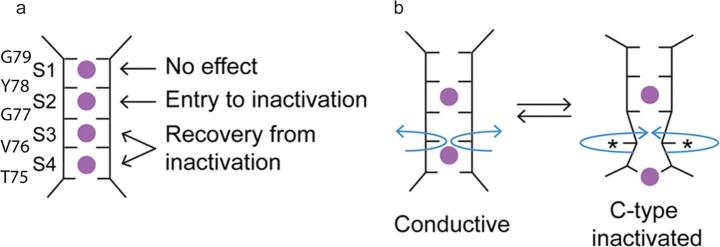
Another scheme proposed to explain the inactivation process. (a) Various sites at the selectivity filter have been shown to be important for inactivation or recovery from inactivation. (b) In this hypothesis, ion binding at the S2 site leads to the deformation at the S3 site, blocking the ion flow and causing inactivation. Adapted from reference (Matulef et al., 2016).

Given the controversy described above, Valiyaveetil and coworkers proposed a novel hypothesis to explain C-type inactivation based on the observation on several semi-synthesized KcsA channels in which amide-to-ester substitution in the protein backbone at certain sites of the selectivity filter was introduced (Matulef et al., 2013). In this hypothesis, the inactivation is linked to ion occupancy at the specific sites in the selectivity filter. (Fig. 8)

When the substitutions are made at residues involved in the S2 binding site ligands (i.e. Y78) or S3 (i.e. G77), they discovered new phenomena. These substitutions slowed the inactivation process, and moreover, crystal structures of these two semi-synthesized KcsA samples show little ion occupancy at the S2 site (Matulef et al., 2013). Analogous phenomena have been observed for Rb^+^ permeating through wild type KcsA channels. Rb^+^ binding slows down the inactivation rates and much reduced ion density is in the S2 site in the crystal structure (Lockless et al., 2007). However, when the substitution site involves the ligands of S1 (i.e. G79), the channel inactivates and in the crystal structure, ion occupancy at S1 is considerably reduced, but ion occupancies at S2-S4 are similar to those in WT. They further introduced perturbations to ion binding at the S3 and S4 sites by applying amide-to-ester substitutions at V76 and T75G mutation respectively (Matulef et al., 2016). These perturbations do not affect the inactivation rate, but strongly prolong the recovery process. Based on this evidence, the authors proposed that the inactivation is caused by local structural distortions in and near V76, which happens when S2 is bound with an ion; this distortion increases the barrier for ions to conduct. Meanwhile, amide-to-ester substitutions do not prevent the selectivity filter from entering the collapsed conformation. The reduction of inactivation by amide-to-ester substitution, on the other hand, appears to disprove the hypothesis that inactivation is caused by the constricted selectivity filter.

The application of semisynthesized protein to perturb ion binding at a specific location greatly expands our understanding of the importance role of ion binding on inactivation. It highlights the importance of ion occupancy in the channel rather than peptide conformation per se. Meanwhile a concern regarding these studies is that the introduction of amide-to-ester substitutions also causes other distortions in the hydrogen-bonding network behind the selectivity filter, which involves the buried water and backbone amide. Valiyaveeti *et al* reasoned that it is not important, as those substitutions do not cause any appreciable structural changes in the selectivity filter or the surrounding residues, and the critical E71–D80 interaction is maintained. Also, the S2 and S3 ester substitutions disrupt different hydrogen bonds, but have similar effects on ion occupancy and inactivation.

To add to the controversy, an MD simulation conducted by Li et al shows that amide-to-ester substituted proteins do transmit from the conductive conformation to the collapsed conformation (Li et al., 2017). The G77 ester, due to the lack of amide to form hydrogen bonding with the buried water, destabilizes the stability of the constricted conformation. This would also prevent the inactivation. The reduction of ion occupancy could be simply caused by ester substitution, as shown in other sites, and may be irrelevant to inactivation. The absence of Rb^+^ at the S2 site can be explained by the observation that the S2 site is the most selective site for K^+^ in the selectivity filter (Roux, 2005). The reduction of inactivation is hypothesized to be caused by the long residence of ions in the selectivity filter, preventing the structure from becoming constricted, which is termed a “ foot in the door” mechanism. Meanwhile, a recent Isothermal Titration Calorimetry (ITC) measurement shows that Rb^+^ binds to KcsA at pH 4 with a higher affinity than K^+^ ions, indicating that Rb^+^ could stabilize the conductive conformation at the activated condition (Liu et al., 2015). We expect that more experiments with suitable time and atomic resolution such as 2D-IR (Kratochvil et al., 2016) are needed to clear the controversy.

### 2.3 Ion dilation hypothesis

**Fig. 9:**
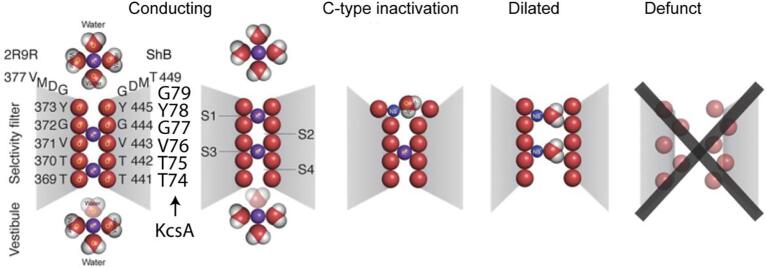
The ion dilation hypothesis proposed by Armstrong et al. to explain the inactivation process in Shaker channel. The C-type inactivation starts with a local deformation at the outer mouth of the selectivity filter, which blocks the ion flow. After the potassium ions are depleted from the selectivity filter, the channel dilates and allows partially dehydrated sodium ions to flow through the channel. The channel eventually enters a defunct state, in which the selectivity filter is in a collapsed conformation. Adapted from reference (Hoshi and Armstrong, 2013).

The so-called ion dilation hypothesis was proposed in the context of studies of voltage-activated potassium channels (Hoshi and Armstrong, 2013) (Fig. 9). In this hypothesis, C-type inactivation is caused by expansion of the outermost site of the selectivity filter from the perfect oxygen cage structure seen in the resting state, reducing its selectivity for binding K^+^. Partially hydrated Na^+^ ions could likely bind to the site with high affinities, and prevent K^+^ ions from entering the selectivity filter and conducting. With bound K^+^ diffusing out of the selectivity filter, the selectivity filter would further dilate from the top to the bottom, allowing permeation of partially dehydrated Na^+^. This explains the increasing conductivity for Na^+^ in the C-type inactivation state (McCoy and Nimigean, 2012). One strong support for this hypothesis is the antagonistic effect of high [K^+^] on C-type inactivation, which is also used to support the activation coupled inactivation hypothesis as discussed above. It is a challenging task to pinpoint the effects of K^+^ on the stabilization of the conductive conformation of the selectivity filter or the prevention of channel dilation, or both, which make it difficult to distinguish the two hypotheses.

Mutation of residue T449 (Y82 in KcsA) in Shaker channels, located at the outer mouth, has shown a significant effect on inactivation. The interaction between T449 and selectivity filter residue Y445 (Y78 in KcsA) and pore helix residue W434 (W67 in KcsA) in Shaker is assumed to be critical for channel dilation. When mutations cause more space for Y445 to rotate and predispose its carbonyl to recede from the pore axis, they lead to channel dilation and increase C-type inactivation. Such mutations include W434F and T449 (A, C, K, E, S) (Hoshi and Armstrong, 2013). Mutations on KcsA at equivalent site (Y82A) also shows enhanced inactivation (Cuello et al., 2017). In contract, when mutations contain bulky side chain and restrict the motion of Y445, the inactivation is abolished. Such mutations are T449 (Y V I) (Hoshi and Armstrong, 2013). It seems difficult to conceive of more direct tests support to such a hypothesis, apart from mutations that could induce global structural changes in the selectivity filter. Notably, a high-resolution Y82A KcsA crystal structure shows a constricted conformation at the selectivity filter, similar to the collapsed conformation (i.e. 1K4C) acquired in the presence of low [K^+^] (Cuello et al., 2017). Interestingly, contrary to the dilation hypothesis, Heer et al show that certain extent of channel dilation is actually necessary for the channel to efficiently conduct [K^+^] (Heer et al., 2017).

It is known that outer mouth sites are critical in the C-type inactivation (Fig. 1). Yellen et al introduced a mutation to a cysteine residue at the T449 site in Shaker channel, showing that Cd^2+^ binding significantly enhanced the inactivation process. Cd^2+^ stabilizes the inactivated conformation, since its affinity is tighter by four orders of magnitude in the inactivated state than in the deactivated state (Yellen et al., 1994). They further introduced cysteine mutations at other outer mouth sites and measured state-dependent changes in accessibility to chemical modification; the modification rates were significantly enhanced in the inactivated state, indicating that conformational changes at the outer mouth occur during C-type inactivation, rendering those residues accessible to modification reagents (Liu et al., 1996). Similar results of Cd^2+^ binding experiments were achieved at the KcsA channel by introducing cysteine mutation at equivalent site (Y82C) by Perozo et al. (Raghuraman et al., 2012). By introducing cysteine mutations at various sites, including double mutations at adjacent and diagonal sites in the tetramer in KcsA and Shaker channel, they demonstrated that Cd^2+^ affects inactivation mainly by cross-linking cysteine in the adjacent sites, implying that the outer mouth loops were constricted to allow Cd^2+^ to bind during inactivation. EPR measurements on radical distances at Y82 sites confirmed that the diagonal distance is shortened from 12 Å to 8 Å during inactivation. These results suggest a closer distance between monomers, rather than dilation. In contrast, comparison of crystal structures between the conductive conformation (1K4C) and the collapsed conformations (1K4C or 3F5W) does not show such a dramatic conformation change in the outer vestibule. This discrepancy is assumed to be caused by the difference in the dynamic behaviors of the outer vestibule, or perhaps by the differences between conditions of X-ray crystallography and EPR (Raghuraman et al., 2014).

Three hypotheses have been introduced above. The major disputed point that distinguishes these hypotheses is the conformation of the selectivity filter in the C-type inactivated state. In the first hypothesis, the collapsed structure, seen in crystal structures, is regarded as the inactivated structure. In the other two hypotheses, conformational change at different local sites in the selectivity filter is suggested and the collapsed state is treated as a ‘deeply inactivated state’ or ‘defunct’ state (Devaraneni et al., 2013, Hoshi and Armstrong, 2013). From a functional perspective, the requirements for the conformation of the selectivity filter for K^+^ conduction indicates that the conformational change in all three hypotheses could prevent K^+^ from conducting. Because many factors such as K^+^ concentration and various mutations can cause coupled changes in the structure and energetics, it may be difficult to isolate one variable to definitively test these hypotheses and distinguish one hypothesis from the other.

From a kinetics perspective, the conformation changes in the last two hypotheses contain two processes: the first is a small distortion of the selectivity filter, and the second is an overall structural collapse. At this point, no crystal structures have been reported corresponding to the proposed initial step, which suggests that this structure might be not thermodynamically stable. Current traces in patch-clamp experiments indicate that C-type inactivation is a seconds-timescale single-exponential decay process for KcsA and many other potassium channels. The recovery from inactivation is even slower, also on a seconds-timescale. For these latter two hypothesis, the inactivation kinetics might be expected to be significantly faster than that in the activation-coupled inactivation hypothesis, since the energetic barrier for a global structural change hypothesized in the activation coupled inactivation hypothesis would likely be larger than that for a local structural change (such as is proposed in the later two hypothesis). The fact that inactivation is a slow (seconds-timescale) process therefore seems to favor the first hypothesis as the main inactivation process, while the local structural change, as suggested in the latter two hypothesis, might exist as initial process in inactivation with a faster dynamics. This might help to explain features displayed in single channel recording current traces, where channels exhibit a “flicker mode” with current constantly flickering on and off before entering a long non-conducting state (Chakrapani et al., 2007a). The flicker behavior has been studied by a recent SSNMR study; together with MD simulation, it shows that the flicker mutant E71Q, comparing to WT, shows more dynamics at the selectivity and higher exchange rate between inward-facing and outward-facing conformation at the V76 carbonyl site, which has been hypothesized to be critical in inactivation in the ion-binding hypothesis (Fig. 8b) (Jekhmane et al., 2019). However, clearly more rigorous measurements on protein dynamics are needed to test such a hypothesis.

## 3 Importance of lipids on potassium channel and inactivation

The importance of lipids on membrane protein stability, structure and function has been discovered in numerous studies (Laganowsky et al., 2014, Lee, 2011, Williamson et al., 2003).The mechanisms of such effects have been generally viewed from two points of view. One aspect is the affect of lipids on membrane protein function through a general physical effect; these effects may includes membrane fluidity, curvature, lateral pressure, and hydrophobic match between lipid and protein. Another aspect is specific binding of lipids on proteins (i.e. annular sites), akin to ligand binding effects, which may stabilize specific conformations, induce protein oligomerization, or otherwise affect function. It is also suggested that lipids per se can have activating or deactivating effects, such as in the case of the sarcoendoplasmic reticulum Ca^2+^-ATPase (Gustavsson et al., 2011). A tremendous number of studies and reviews have focused on these effects for various proteins (Brohawn et al., 2012, Ketchem et al., 1993, Lee, 2011, Sharma et al., 2010, van Dalen et al., 2002). Potassium channels are good examples in this sense, and are extensively studied in term of protein-lipid interaction. For voltage gated potassium channels, interactions between the phospholipids and voltage sensing residues such as arginine are crucial for channel activation (Thomson and Rothberg, 2010, Zheng et al., 2011). Other membrane components such as cholesterol have also been shown to influence the channel’s functions (Cornelius et al., 2015, Delgado-Ramírez et al., 2018, Lingwood and Simons, 2010).

In KcsA channels, anionic lipids have been found co-crystalized with the protein and also have been identified in SSNMR experiments binding at grooves between transmembrane helices, i.e. monomer–monomer interface (Weingarth et al., 2013, Zhou et al., 2001). Residues R64 and R89 have been identified as the key residues interacting with the negatively charged lipid headgroup. These interactions have been shown to be critical not only for stabilizing the tetrameric form of the channel, but also possibly for modulating channel inactivation and open probabilities (Raja, 2011). Lee et al showed that by increasing the anionic lipid content (such as PS, PA) of the membrane, the channel’s open probability is greatly increased (Marius et al., 2008). They hypothesized that the anionic lipids that are bound to the protein help stabilize the conductive conformation of the selectivity filter and inhibit the inactivation process. Similar effects can be achieved by introducing cardiolipin, which is also more negatively charged, into the lipid bilayer. (van der Cruijsen et al., 2017).

By contrast, other studies indicate that anionic lipids bound at annular sites could cause conformational changes at the back of the selectivity filter, facilitating the W67-E71-D80 hydrogen-bond network, which is known to cause the channel to inactivate (Poveda et al., 2017). The authors reasoned that when anionic lipids are bound, they interact with R89 and R64, which render W67 and D80 able to form hydrogen bonds with E71. In contrast, when there is no lipid bound, the proteins tend to dock on each other to from a cluster while R89 and R64 would, instead, form salt bridges with D80 W67 residues, weakening the W67-E71-D80 hydrogen-bond network and inhibits channel inactivation. By mutating the arginine residues to alanine, they increased the likelihood of the protein to form clusters, and increased the channel open probabilities as well, as shown in single channel recording experiments (Molina et al., 2015). These apparently contradictory effects will be important to pursue further. Clustering behavior of KcsA has been confirmed by SSNMR experiments in which singly labeled KcsA was prepared with ^13^C or ^15^N isotopes and then mixed. ^13^C-^15^N polarization transfers occur for inter-tetramer contacts within 5 Å (Visscher et al., 2017).

Lipid bilayers have been prepared in asymmetric fashion so that the effects on the channels function of lipids in inner vs. outer membranes could be distinguished (Iwamoto and Oiki, 2013a, Iwamoto and Oiki, 2013b). These experiments focused on E71A mutants, which have a high open probability; this choice provided experimental convenience for current recording and data analysis. By comparing the channel open probabilities in various conditions in terms of including negatively or positively charged lipids in the inner or outer membrane, they concluded that negatively charged lipids in the inner membrane can interact nonspecifically with the positive charges in the N-terminal helix (residues 1–25) after the channel is activated and stabilized the activated pH-gate conformation, which increases the channel open probability in the inactivation less E71A channel. The authors did not discover influence of lipids in the outer membrane on channel function, which again appears to be contradictory to the studies we discussed above. However, since the W68-E71-D80 hydrogen-bond network is impaired in E71A and the non-annular lipid seems affect the channel through this network, it maybe reasonable to not observe an effect of lipids in the outer membrane on the mutant channel function. Perhaps in WT channels, the stabilization effect on the open pH-gate conformation from negatively charged lipids could allosterically destabilize the selectivity filter and thus enhance the inactivation in WT and prolong the recovery of the channel from the inactivated state. Such a hypothesis appears to be supported by the experiments they report on the WT, suggesting that adding negatively charged lipids in the inner membranes actually slightly decreased the channel open probability for WT.

Fewer studies of the influence of lipids on channel function via physical (mechanical, dimensional) effects have been documented, possibly because these experiments are generally more difficult to quantify. Rusinova et al showed that the phospholipid acyl chain length could perturb KcsA function primarily by changes in bilayer thickness rather than changes in the local lipid composition around channels (Rusinova et al., 2014). We hypothesize that the allosteric coupling strength could also be modulated by the physical properties of the membrane; thus the inactivation process is affected, but these aspects remain to be tested.

## 4 Perspectives

We summarized current views on inactivation study and as we can see, manipulating ion affinities, which is beneath each hypothesis, is a powerful strategy for controlling channel activities. A large portion of these researches on potassium channels has been carried out on KcsA due to the availability of crystal structures under a terrific range of conditions, as well as the ease of sample preparation, functional characterization, and the relative ease of computation. KcsA has proven to be a good model to study the permeation problem and share a general scheme on how the channel is activated; here we retrieve the question whether KcsA is also a good model for studying inactivation in potassium channels and the answer to this question is quite complicated.

While it is an essentially universal property of potassium channels, there are a great variety of kinetic profiles in the inactivation seen in potassium channels. For example, hERG channels show faster inactivation processes as compared to that in Shaker or KcsA (Wang and MacKinnon, 2017). Some potassium channels do not inactivate to a complete extent. One example is the calcium-gated MthK channel, which shows relatively little inactivation in the lipid bilayer (Thomson and Rothberg, 2010). Such diversity is probably rooted in the protein sequence. As shown in the sequence alignments (Fig. 2), many critical residues for inactivation in KcsA are not fully conserved in other channels, for example the critical E71 residue. Furthermore, in voltage-gated channels inactivation is voltage dependent. The voltage sensor apparently is coupled to the selectivity filter during the inactivation process (Sadovsky and Yifrach, 2007, Thomson and Rothberg, 2010). Interestingly, KcsA (lacking a voltage sensor) also shows voltage dependent inactivation, which has been attributed to voltage modulation of the strength of the E71-D80 hydrogen bonding interaction (Cordero-Morales et al., 2006). In other words, this is presumed to be a distinct process from the voltage modulation in voltage gated channels.

Overall, though, evidence from many studies shows that the molecular machinery identified in KcsA provides valuable information for other potassium channels on inactivation. Mutation profiles have analogous effects on inactivation for KcsA and voltage gated channels, even though many of the critical residues in inactivation in KcsA are not strictly conserved. For example, Kv1.2 the Shaker K^+^ channel family shows less C-type inactivation (than KcsA) after the N-type inactivation is removed, and it has a valine in the position equivalent to the E71 in KcsA. Mutation of E71 to valine in KcsA significantly reduces the inactivation, which is assumed to break the E71-D80-W67 interaction for inactivation. By introducing a V370E mutation in Kv1.2, which can interact with D379 (analogous to D80 in KcsA), the C-type inactivation behavior was greatly enhanced in the mutant channel (Cuello et al., 2010b). Similarly, in the Shaker channel, by mutating the I470 (F103 in KcsA) to residues with smaller side chains such as alanine and cysteine, the C-type inactivation is greatly reduced, which mirrors the effect of mutating F103 to smaller side chain residues in KcsA (Cuello et al., 2010a). Other examples can be found in the positions such as E51, D80, and Y82 (Cuello et al., 2017, Hoshi and Armstrong, 2013, van der Cruijsen et al., 2013). Therefore, we hypothesize that even though residues for C-type inactivation in the potassium channel have been constantly evolving and modulated to cope with the evolutionary pressure, the basic mechanism is shared in among potassium channels, making KcsA a valuable model for studying inactivation. For the future study, focus on the deviation on functional characteristics in various channels will be interesting and rewarding since it will be a huge leap for our understanding of molecular mechanisms behind each channel and beneficial for drug development as well as protein design in general.

SSNMR has been shown an insightful tool for studying ion channels. As we have argued above, lipid environments have been a crucial factor for ion channels to function properly. The ability of SSNMR to provide information on ligand or ion affinities, protein conformational changes and water/lipid-protein interactions in an authentic environment makes it quite a unique tool for channel studies. One great advantage of NMR study is the wide spectrum of dynamics information it can measure. However, it is less well exploited in current channel study mainly due to the difficulty in preparing suitable sample conditions and spectra assignments as well as a lack of suitable NMR pulse sequences. But we believe that there will be more NMR dynamics study in the near future, which combines with molecular dynamic simulation to provide a vivid video for the mechanisms of channel functions.

## Declaration of Competing Interest

The authors declare no conflict of interest with respect to this publication.
